# Conceptualizing the complexity of reflective practice in education

**DOI:** 10.3389/fpsyg.2022.1008234

**Published:** 2022-10-19

**Authors:** Misrah Mohamed, Radzuwan Ab Rashid, Marwan Harb Alqaryouti

**Affiliations:** ^1^Centre for Enhancement of Learning and Teaching, University of West London, London, United Kingdom; ^2^Faculty of Languages and Communication, Universiti Sultan Zainal Abidin, Terengganu, Malaysia; ^3^Department of English Language, Literature and Translation, Zarqa University, Zarqa, Jordan

**Keywords:** criticality, reflection, revised model, reflective practice, problematizing

## Abstract

In higher education, reflective practice has become a dynamic, participatory, and cyclical process that contributes to educators’ professional development and personal growth. While it is now a prominent part of educators, many still find it challenging to apply the concept for it carries diverse meaning for different people in different contexts. This article attempts to (re)conceptualize the complexity of reflective practice in an educational context. Scholars in this field have taken different approaches to reflective practice, but all these approaches consist of four main components in common: (i) reflecting; (ii) planning for future action; (iii) acting; and (iv) evaluating the outcomes. We extend the existing literature by proposing a model which integrates these four components with three key aspects of reflection: problem-solving, action orientation, and criticality. The novelty of this model lies within its alignment of the three key aspects with different levels of criticality in a comprehensive framework with detailed descriptors provided. The model and its descriptors are useful in guiding individuals who directly or indirectly involve in critical reflection, especially educators, in appraising their levels of criticality and consequently engage in a meaningful reflection.

## Introduction

In the field of education, reflective practice has been recognized as an important aspect in continuing professional development. Through reflective practice, we can identify the factors, the consequences of and the assumptions that underlie our actions. In higher education, reflective practice has become a dynamic, participatory, and cyclical process (Ai et al., 2017) that contributes to educators’ professional development and personal growth (McAlpine et al., 2004; De Geest et al., 2011; Davies, 2012; Marshall, 2019). It enables professional judgment (Day, 1999) and fosters professional competence through planning, implementing and improving performance by rethinking about strengths, weaknesses and specific learning needs (Huda and Teh, 2018; Cirocki and Widodo, 2019; Zahid and Khanam, 2019; Seyed Abolghasem et al., 2020; Huynh, 2022). Without routinely engaging in reflective practice, it is unlikely that educators will comprehend the effects of their motivations, expectations and experiences upon their practice (Lubbe and Botha, 2020). Thus, reflective practice becomes an important tool that helps educators to explore and articulate lived experiences, current experience, and newly created knowledge (Osterman and Kottkamp, 2004). Educators are continually recommended to apply reflective practice in getting a better understanding of what they know and do as they develop their knowledge of practice (Loughran, 2002; Lubbe and Botha, 2020). In fact, reflective practice is now a prominent part of training for trainee teachers (e.g., Shek et al., 2021; Childs and Hillier, 2022; Ruffinelli et al., 2022) because it can help future teachers review their own practices and develop relevant skills where necessary.

Despite the wide acceptance of the concept of reflective practice, the notion of ‘reflection’ in itself is still broad. Our review of literature reveals that reflection is a term that carries diverse meaning. For some, “it simply means thinking about something” or “just thinking” (e.g., Loughran, 2002, p. 33), whereas for others, it is a well-defined practice with very specific purpose, meaning and action (e.g., Dewey, 1933; Schön, 1983; Grimmett and Erickson, 1988; Richardson, 1990; Loughran, 2002; Spalding et al., 2002; Paterson and Chapman, 2013). We found many interesting interpretations made along this continuum, but we believe the most appealing that rings true for most people is that reflection is useful and informing in the development and understanding of teaching and learning (e.g., Seitova, 2019; McGarr, 2021; Huynh, 2022). This, however, is not enough to signify the characteristics of reflection. Consequently, many teachers find it hard to understand the concept and engage in reflective practice for their professional development (Bennett-Levy and Lee, 2014; Burt and Morgan, 2014; Haarhoff et al., 2015; Marshall, 2019; Huynh, 2022; Knassmüller, 2022; Kovacs and Corrie, 2022). For example, some teachers from higher arts education have considered reflective practice as antithetical to practical learning (Guillaumier, 2016; Georgii-Hemming et al., 2020) as they often frame explicit reflection as assessed reflective writing, which is “disconnected from the embodied and non-verbal dimensions of making and reflecting on art” (Treacy & Gaunt, 2021, p. 488). The lack of understanding of the concept has created disengagement in reflection and reflective practice (Aliakbari and Adibpour, 2018; Huynh, 2022; Knassmüller, 2022) which resulted in poor insight and performance in practice (Davies, 2012). To overcome this, educators should foster their understanding of the reflective practice, so they not only can reap its benefits for their own learning, but also facilitate and maximize reflective skills within their students.

In this paper, we aim to provide an overview of the concepts of effective reflective practice and present the value of reflective practice that can help teachers to professionally develop. First, we situate our conceptual understanding of reflective practice by discussing key issues surrounding reflection and reflective practice. Second, we present the key aspects of effective reflective practice. Finally, based on our discussion of key aspects of effective reflective practice, we introduce a revised model of reflective practice that may serve as a guide for educators to professionally develop. Although the model is but one approach, we believe it holds promise for others grappling as we are with efforts to encourage reflective practices among educators who find reflection in and on their practices a complex concept.

## Key issues in reflective practice

The concepts of “reflection,” “reflective thought,” and “reflective thinking” have been discussed since 1904, when John Dewey claimed that an individual with good ethical values would treat professional actions as experimental and reflect upon their actions and consequences. Dewey defined reflection as the “active, persistent, and careful consideration of any belief or supposed form of knowledge in the light of the grounds that support it and the further conclusions to which it tends” (Dewey, 1904, p. 10). His basic notion is that reflection is an active, deliberative cognitive process involving a sequence of interconnected ideas that include the underlying beliefs and knowledge of an individual.

Following Dewey’s original work and its subsequent interpretation, four key thought-provoking issues are worthy of discussion: reflective thinking versus reflective action; time of reflection; reflection and problem solving; and critical reflection. The first concern is whether reflection is a process limited to thinking about action or also bound up in action (Grant and Zeichner, 1984; Noffke and Brennan, 1988; Hatton and Smith, 1995). There seems to be broad agreement that reflection is a form of thought process (Ross, 1989; McNamara, 1990; Sparks-Langer et al., 1991; Hatton and Smith, 1995) even though some do not lead to action. However, Dewey’s first mention of “reflective action” suggests he was concerned with the implementation of solutions after thinking through problems. Therefore, reflective practice, in our view, is bound up with the constant, careful consideration of practice in the light of knowledge and beliefs. The complete cycle of reflection should then lead to clear, modified action and this needs to be distinguished from routine action derived from impulse, tradition, or authority (Noffke and Brennan, 1988; Gore and Zeichner, 1991; Hatton and Smith, 1995).

The time frames within which reflection takes place, needs to be addressed—relatively immediate and short term, or rather more extended and systematic. Schön (1983) holds that professionals should learn to frame and reframe the problems they often face and after trying out various interpretations, modify their actions as a result. He proposes “reflection-in-action,” which requires conscious thinking and modification, simultaneously reflecting and doing almost immediately. Similar to this concept is “technical reflection,” involving thinking about competencies or skills and their effectiveness and occurs almost immediately after an implementation and can then lead to changes in subsequent action (Cruickshank, 1985; Killen, 1989). While the notion of immediacy in reflective practice seems appropriate, some argue that the process should involve conscious detachment from an activity after a distinct period of contemplation (Boud et al., 1985; Buchmann, 1990). This is because reflection demands contemplating rational and moral practices in order to make reasoned judgments about better ways to act. Reflective practice often involves looking back at actions from a distance, after they have taken place (Schön, 1983; Gore and Zeichner, 1991; Smith and Lovat, 1991). While immediate and extended “versions” of reflections are both recognized, we suppose no one is better than another. However, we believe that being able to think consciously about what is happening and respond instantaneously makes for a higher level of reflective competence.

The third issue identified from our literature review is whether reflection by its very nature is problem orientated (Calderhead, 1989; Adler, 1991). Reflection is widely agreed to be a thought process concerned with finding solutions to real problems (Calderhead, 1989; Adler, 1991; Hatton and Smith, 1995; Loughran, 2002; Choy and Oo, 2012). However, it is unclear whether solving problems is an inherent characteristic of reflection. For example, Schön’s (1983) reflection-in-action involves thought processing simultaneously with a group event taking place, and reflection-on-action refers to a debriefing process after an event. Both aims to develop insights into what took place—the aims, the difficulties during the event or experience and better ways to act. While focusing on reacting to practical events, these practices do not often intend to find solutions to specific practical problems. Instead, reflective practitioners are invited to think about a new set of actions from if not wider, at least different perspectives.

The fourth issue in the literature revolves around “critical reflection.” Very often critical reflection is concerned with how individuals consciously consider their actions from within wider historical, cultural and political beliefs when framing practical problems for which to seek solutions (Gore and Zeichner, 1991; Hatton and Smith, 1995; Choy and Oo, 2012). It is a measure of a person’s acceptance of a particular ideology, its assumptions and epistemology, when critical reflection is developed within reflective practice (McNamara, 1990; Hatton and Smith, 1995). It implies the individual locates any analysis of personal action within her/his wider socio-historical and political-cultural contexts (Noffke and Brennan, 1988; Smith and Lovat, 1991; Hatton and Smith, 1995). While this makes sense, critical reflection in the literature appears to loosely refer to an individual’s constructive self-criticism of their actions to improve in future (Calderhead, 1989), not a consideration of personal actions with both moral and ethical criteria (Senge, 1990; Adler, 1991; Gore and Zeichner, 1991). Thus, we see a need to define critical reflection in line with the key characteristics of reflective practice.

## Effective reflective practice

Reflecting on the issues discussed above, we conclude that for reflective practice to be effective, it requires three key aspects: problem-solving, critical reflection and action-orientation. However, these aspects of reflective practice have different levels of complexity and meaning.

### Problem-solving

A problem is unlikely to be acted upon if it is not viewed as a problem. Thus, it is crucial to problematize things during reflection, to see concerns that require improvement. This is not a simple process as people’s ability to perceive things as problems is related to their previous experiences. For example, a senior teacher with years of teaching experience and a rapport with the students s/he teaches will be immediately aware of students experiencing difficulties with current teaching strategies. However, a junior teacher whose experience is restricted to a three-month placement and who has met students only a few times will be less aware. The differences in experience also influence the way people interpret problems. For example, the senior teacher may believe his/her teaching strategy is at fault if half the students cannot complete the given tasks. A junior teacher with only 2 weeks teaching experience may deduce that the students were not interested in the topic, and that is why they cannot complete the tasks given. This example illustrates the range of ways a problem can be perceived and the advantages of developing the ability to frame and reframe a problem (Schön, 1983). Problems can also be perceived differently depending on one’s moral and cultural beliefs, and social, ethical and/or political values (Aliakbari and Adibpour, 2018; Karnieli-Miller, 2020). This could be extended to other factors such as institutional, educational and political system (Aliakbari and Adibpour, 2018).

Framing and reframing a problem through reflection can influence the practice of subsequent actions (Loughran, 2002; Arms Almengor, 2018; Treacy and Gaunt, 2021). In the example above, the junior teacher attributes the problem to the students’ attitude, which gives her/him little to no incentive to address the situation. This is an ineffective reflective practice because it has little impact on the problem. Thus, we believe it is crucial for individuals to not only recognize problems but to examine their practices (Loughran, 2002; Arms Almengor, 2018; Zahid and Khanam, 2019) through a different lens to their existing perspectives so solutions can be developed and acted upon. This requires critical reflection.

### Critical reflection

We believe it is the critical aspect of reflection that makes reflective practice effective and more complex, formulated by various scholars as different stages of reflection. Zeichner and Liston (1987) proposed three stages of reflection similar to those described by Van Manen (1977). They suggested the first stage was “technical reflection” on how far the means to achieve certain end goals were effective, without criticism or modification. In the second stage, “practical reflection,” both the means and the ends are examined, with the assumptions compared to the actual outcomes. This level of reflection recognizes that meanings are embedded in and negotiated through language, hence are not absolute. The final stage, “critical reflection,” combined with the previous two, considers both the moral and ethical criteria of the judgments about professional activity (Senge, 1990; Adler, 1991; Gore and Zeichner, 1991).

While the three stages above capture the complexity of reflection, individuals will only reach an effective level of reflection when they are able to be self-critical in their judgments and reasoning and can expand their thinking based on new evidence. This aligns with Ross’ (1989) five stages of reflection (see Table 1). In her five stages of reflection, individuals do not arrive at the level of critical reflection until they get to stages 4 and 5, which require them to contextualize their knowledge and integrate the new evidence before making any judgments or modification (Van Gyn, 1996).

**Table 1:** Five stages of reflections (Ross, 1989).

	The individual
Stage 1	Has a simple view of the world Believes knowledge to be absolute Views authority as the source of knowledge
Stage 2	Acknowledges existence of different viewpoints Believes knowledge to be relative Sees varying positions between right or wrong, no absolutes Uses unsupported personal beliefs frequently as “hard” evidence when making decisions Views truth as “knowable” but not yet known
Stage 3	Perceives legitimate differences of viewpoint Begins to develop the ability to interpret evidence Uses unsupported personal belief and evidence in making decisions but is beginning to be able to differentiate between them Believes that knowledge is uncertain in some areas
Stage 4	Views knowledge as contextually based Develops views that an integrated perspective can be evaluated as more or less likely to be true Develops an initial ability to integrate evidence to develop a coherent point of view
Stage 5	Exhibits all the characteristics listed in Stage 4 Possesses the ability to make objective judgments based on reasoning and evidence Is able to modify judgments based on new evidence if necessary

### Action-orientation

We believe it is important that any reflections should be acted upon. Looking at the types and stages of reflection discussed earlier, there is a clear indication that reflective practice is a cyclical process (Kolb, 1984; Richards and Lockhart, 2005; Taggart and Wilson, 2005; Clarke, 2008; Pollard et al., 2014; Babaei and Abednia, 2016; Ratminingsih et al., 2018; Oo and Habók, 2020). Richards and Lockhart (2005) suggest this cyclical process comprises planning, acting, observing, and reflecting. This is further developed by Hulsman et al. (2009) who believe that the cyclical process not only involves action and observation, but also analysis, presentation and feedback. In the education field, reflective practice is also considered cyclical (Clarke, 2008; Pollard et al., 2014; Kennedy-Clark et al., 2018) because educators plan, observe, evaluate, and revise their teaching practice continuously (Pollard et al., 2014). This process can be done through a constant systematic self-evaluation cycle (Ratminingsih et al., 2018) which involves a written analysis or an open discussion with colleagues.

From the descriptions above, it seems that cyclical reflective practice entails identifying a problem, exploring its root cause, modifying action plans based on reasoning and evidence, executing and evaluating the new action and its results. Within this cyclical process, we consider action as a deliberate change is the key to effective reflective practice, especially in the field of education. Reflection that is action-oriented is an ongoing process which refers to how educators prepare and teach and the methods they employ. Educators move from one teaching stage to the next while gaining the knowledge through experience of the importance/relevance of the chosen methods in the classroom situation (Oo and Habók, 2020).

## Discussion

**Figure 1:**
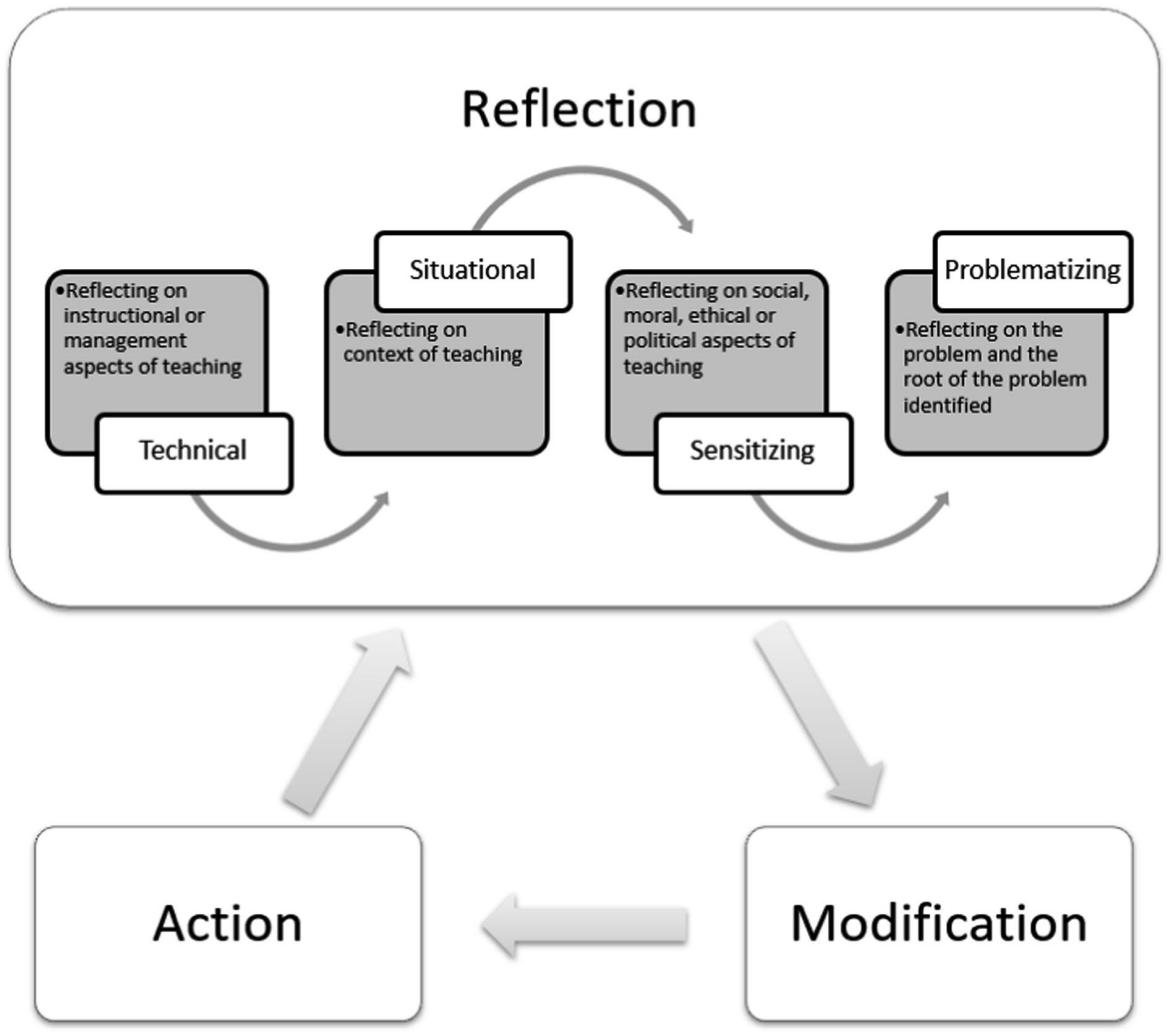
Cyclical reflective practice model capturing problem-solving, action-oriented critical reflection.

While reflection is an invisible cognitive process, it is not altogether intuitive (Plessner et al., 2011). Individuals, especially those lacking experience, may lack adequate intuition (Greenhalgh, 2002). To achieve a certain level of reflection, they need guidance and this can be done with others either in groups (Gibbs, 1988; Grant et al., 2017) or through one-on-one feedback (Karnieli-Miller, 2020). The others, who can be peers or mentors, can help provide different perspectives in exploring alternative interpretations and behaviors. Having said this, reflecting with others may not always feasible as it often requires investment of time and energy from others (Karnieli-Miller, 2020). Therefore, teachers must learn how to scaffold their own underlying values, attitudes, thoughts, and emotions, and critically challenge and evaluate assumptions of everyday practice on their own. With this in mind, we have created a cyclical process of reflective practice which may help in individual reflections. It captures the three key aspects of reflective practice discussed above. This model may help teachers having a range of experience enhance their competence through different focus and levels of reflection (see Figure 1).

The model illustrates the cyclical process with three stages: reflection, modification and action. At the reflection stage, a problem and the root of the problem is explored so it can be framed as it is/was and then reframed to identify a possible solution. This is followed by a modification for change based on the reasoning and evidence explored during the reflection stage. Finally, the action stage involves executing action (an event), followed by the reflection stage to begin another cycle and continue the process.

As presented earlier, it is crucial for individuals to be able to frame and reframe problems through a different lens to their existing perspectives so solutions can be developed and acted upon. Thus, the model above expands Tsangaridou and O’Sullivan’s (1994) framework by adding together the element of problematizing. The current revised framework highlights the four focuses of reflection; technical addresses the management or procedural aspects of teaching practice; situational addresses the context of teaching; sensitizing involves reflecting upon the social, moral, ethical or political concerns of teaching; and problematizing concerns the framing and reframing of the problem identified within the teaching context. Considering the different levels of critical reflection, we extend the four focuses of reflection to three different levels of critical reflection: descriptive involves reflection of the four focuses without reasoning or criticism; descriptive with rationale involves reflection of the four focuses with reasoning; and descriptive with rationale and evaluation involves reflection of the four focuses with both reasoning and criticism (see Table 2). Each of these levels requires different degrees of critical analysis and competence to extract information from actions and experiences. Overall, level three best captures effective critical reflection for each focus.

**Table 2:** A framework of reflection.

Focus level	Technical	Situational	Sensitizing	Problematizing
1 Descriptive	Reflecting on the implementation of teaching by providing descriptive information about an action	Reflecting on the contextual aspects of teaching by providing descriptive information about the environment or situation	Reflecting on any other aspects of teaching by providing descriptive information about social, moral, ethical or political values that underpin an action	Reflecting on areas for development by providing descriptive information about the problem identified
2 Descriptive with rationale	Reflecting on the implementation of teaching by providing descriptive information about an action, and the rationale for an action (why it was carried out)	Reflecting on contextual aspects of teaching by providing descriptive information about the environment or situation, and the rationale for an action (why it was used in that specific context)	Reflecting any other aspects of teaching by providing descriptive information about social, moral, ethical, or political values that underpin an action, and the rationale for an action (concerning either the context or methods used, why decisions were made)	Reflecting on areas for development by providing descriptive information about the problem identified and its root (why the problem occurred)
3 Descriptive with rationale and evaluation	Reflecting on the implementation of teaching by providing descriptive information about an action, the rationale for an action, and evaluation of an action	Reflecting on contextual aspects of teaching by providing descriptive information about the environment or situation, rationale for an action (why it was used in that specific context, and evaluation of an action)	Reflecting on social, moral, ethical or political aspects of teaching by providing descriptive information about social, moral, ethical or political values that underpin an action, and the rationale for an action (concerning either the context or methods used, why decisions were made), and evaluation of implications of an action	Reflecting on areas for development by providing descriptive information about the problem identified and its root (why the problem occurred) and evaluation of the logic underpinning the procedure (reframing problem)

This revised model that we proposed encompasses different levels of critical reflection and is action-oriented. There is also a clear link to problem-solving which requires framing and reframing problems to accurately identify them, which may influence the value and effectiveness of the actions that follow (Loughran, 2002). Thus, this model may help people, especially those with lack experience to recognize the different aspects of reflection so they can make better assessments of and modifications to their procedures (Ross, 1989; Van Gyn, 1996).

## Conclusion

The meaning of reflection and reflective practice is not clear cut. However, we believe a reflective educator should cultivate a set of responses to how their teaching operates in practice. As Dewey (1933) suggested, educators must find time to reflect on their activity, knowledge, and experience so that they can develop and more effectively serve their community, nurturing each student’s learning. However, this does not always happen. Some educators do not reflect on their own practice because they find the concept of reflective practice difficult to put into practice for their professional development (Jay and Johnson, 2002; Bennett-Levy and Lee, 2014; Burt and Morgan, 2014; Haarhoff et al., 2015; Marshall, 2019; Huynh, 2022).

Our review of the literature indicates that reflective practice is a complex process and some scholars argue that it should involve active thinking that is more bound up with action (Grant and Zeichner, 1984; Noffke and Brennan, 1988; Hatton and Smith, 1995). Thus, the complete cycle of reflective practice needs to be distinguished from routine action which may stem from impulse, tradition, or authority (Noffke and Brennan, 1988; Gore and Zeichner, 1991; Hatton and Smith, 1995). In addition, some also argue that reflective practice involves the conscious detachment from an activity followed by deliberation (Boud et al., 1985; Buchmann, 1990), and therefore reflective practice should not occur immediately after action. Although this is acceptable, we believe that instant reflection and modification for future action can be a good indicator of an individual’s level of reflective competence.

Reflective practice is an active process that requires individuals to make the tacit explicit. Thus, it is crucial to acknowledge that reflection is, by its very nature, problem-centered (Calderhead, 1989; Adler, 1991; Hatton and Smith, 1995; Loughran, 2002; Choy and Oo, 2012). Only with this in mind can individuals frame and reframe their actions or experiences to discover specific solutions. Reflective practice is also complex, requiring critical appraisal and consideration of various aspects of thought processes. Individuals must play close attention to what they do, evaluate what works and what does not work on a personal, practical and professional level (Gore and Zeichner, 1991; Hatton and Smith, 1995; Choy and Oo, 2012). However, some would consider critical reflection as no more than constructive self-criticism of one’s actions with a view to improve (Calderhead, 1989). Consequently, scholars have taken different approaches to reflective practice in teaching areas that include critical thinking (e.g., Ross, 1989; Tsangaridou and O’Sullivan, 1994; Loughran, 2002). These approaches had four components in common: reflecting (observing actions, reviewing, recollecting), planning for future action (thinking and considering), acting (practice, experience, and learning), and evaluating (interpreting and assessing outcomes). We propose a model that embraces these four sub-areas and three key aspects of reflection: problem-solving, action orientation and critical reflection. We align these key aspects with level of criticality in a framework with detailed descriptors. It is hoped that these elements, combined together, demonstrate the complexities of reflection in a better, clearer way so that those struggling to adopt reflective practice will now be able to do so without much difficulty.

## Author contributions

MM contributed to conception and written the first draft of the manuscript. RR contributed in the discussion of the topic. All authors contributed to the article and approved the submitted version.

## Conflict of interest

The authors declare that the research was conducted in the absence of any commercial or financial relationships that could be construed as a potential conflict of interest.

## Publisher’s note

All claims expressed in this article are solely those of the authors and do not necessarily represent those of their affiliated organizations, or those of the publisher, the editors and the reviewers. Any product that may be evaluated in this article, or claim that may be made by its manufacturer, is not guaranteed or endorsed by the publisher.
